# Standardized 3D-printed trabecular titanium augment and cup for acetabular bone defects in revision hip arthroplasty: a mid-term follow-up study

**DOI:** 10.1186/s13018-023-03986-0

**Published:** 2023-07-22

**Authors:** Linjie Hao, Yumin Zhang, Weiguo Bian, Wei Song, Kun Li, Nengjun Wang, Pengfei Wen, Tao Ma

**Affiliations:** 1 Department of Joint Surgery, Honghui Hospital, Xi’an Jiaotong University, No. 555 Youyi East Road, Xi’an, 710000 Shaanxi China; 2 Department of Orthopedics, The First Affiliated Hospital of Xi’an Jiaotong University, 277 West Yanta Road, Xi’an, 710061 Shaanxi China

**Keywords:** Standardized 3D‑printed augment, Standardized 3D‑printed cup, Trabecular titanium, Revision, Bone defect

## Abstract

**Background:**

The aim of this study was to assess the feasibility and outcomes of standardized three-dimensional (3D)-printed trabecular titanium (TT) cups and augments to reconstruct most acetabular defects.

**Methods:**

We included 58 patients with Paprosky type II and III acetabular bone defects who underwent revision hip arthroplasty between 2015 and 2018. Patients who were revised without 3D-printed augments, and cases who were lost to follow-up and died during follow-up were excluded. Radiographic and clinical outcomes were evaluated. A Kaplan–Meier survivorship curve was generated. The mean follow-up was 64.5 (range 49–84) months.

**Results:**

In total, 48 (82.8%) acetabular revisions were performed using standardized 3D-printed TT cups and augments, and a retrospective review was conducted on 43 revisions. The average position of the vertical center of rotation and leg length discrepancy were significantly decreased from 42.4 ± 9.1 mm and 38.4 ± 10.7 mm to 22.8 ± 3.4 mm and 4.1 ± 3.0 mm, respectively. Non-progressive radiolucent lines were observed in 3 (7.5%) acetabular components with no indications for revision. The mean Harris hip score, Oxford hip score and EuroQol five-dimensional questionnaire score increased from 33.0 ± 10.7, 11.4 ± 3.4 and 0.29 ± 0.09 to 80.3 ± 8.8, 35.8 ± 2.4 and 0.71 ± 0.10, respectively. The revision-free survival rate of the acetabular component was 93.0% (40/43), with a rate of revision for aseptic loosening of 2.3% (1/43).

**Conclusion:**

Standardized 3D‑printed TT augments and cups could be used to reconstruct the majority of Paprosky type II and III acetabular defects in revision hip arthroplasty and demonstrated encouraging results at mid-term follow-up.

## Introduction

In revision hip arthroplasty, jumbo cup is commonly used for the reconstruction of acetabular bone defects. But it is not always suitable for all severe defects due to unsatisfactory initial stability or insufficient bone-implant contact. According to Johanson et al. [1], although high-grade defects were seen in only 17% of the 1094 revision hips, the failure rate associated with them was 30%. Whether the bone defect can be effectively reconstructed will directly affect the success of the revision.

For the majority of Paprosky type II and III acetabular bone defects, reconstruction with ring, cage, impaction bone grafting (IBG), structural allograft, cup cage and augment are frequently used [2]. Although good initial stability can be obtained by using rings and cages [3, 4], the long-term survivorship is not satisfactory [5, 6]. The use of bone grafts is often limited by the source, as well as the risk of transmitted diseases and bone absorption [7]. The use of cup cages is usually applied to the reconstruction of severe acetabular bone defects with pelvic discontinuity.

Due to the porous trabecular structure and excellent osteointegration capacity, recent studies with three-dimensional (3D)-printed trabecular titanium (TT) augments and trabecular metal (TM) cups have shown encouraging early or mid-term results [8–11]. In our opinion, custom-made 3D-printed cups and standardized 3D-printed augments and cups are all good choices for the treatment of acetabular bone defects. In some cases, using a custom-made cup could avoid the stress between the augment and cup interface and help reproduce the correct center of rotation (COR) [12]. However, custom-made 3D-printed acetabular components with higher costs and longer manufacturing times are more suitable for extremely severe bone defects, and more extensive soft tissue dissection is often required for exposure and fixation. Standardized 3D-printed augments and cups are off the shelf with excellent osteogenic properties, are inexpensive and are convenient to obtain. The aim of this study was to assess the feasibility, survivorship, and radiological and clinical outcomes of standardized 3D-printed augments and cups for the reconstruction of most acetabular defects.

## Materials and methods

### Patients selection

**Fig. 1:**
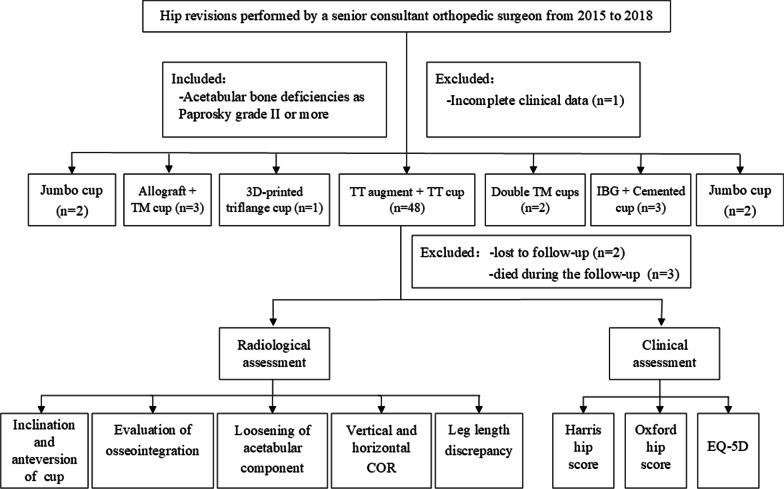
Flowchart detailing the procedure of patients’ selection and follow-up. *TM* Trabecular metal, *3D* three-dimensional, *TT* trabecular titanium, IBG impaction bone grafting, *COR* center of rotation, *EQ-5D* EuroQol five-dimensional questionnaire

In accordance with the ethical standards of the committee on human experimentation of our institution, we retrospectively reviewed our hip revisions performed by a senior consultant orthopedic surgeon from 2015 to 2018. Patients were included if their clinical records and radiographs were consensually graded according to the Paprosky classification [13] of acetabular bone deficiencies as grade II or more. There were 59 total or partial (acetabular only) revisions in 59 patients were selected, and 1 was excluded due to incomplete clinical data. Seven different reconstruction techniques were applied for the remaining 58 patients (24 men and 34 women) (Table 1). The mean age at the time of revision was 61.7 (29–88) years. Of the 48 patients (48 hips) who were treated using standardized 3D‑printed TT cups and augments, 2 patients were lost to follow-up, and 3 died during the follow-up without undergoing further revision. Finally, 43 revisions in 43 patients were included. The patient flow diagram is shown in Fig. 1.

**Table 1:** Relations between Paprosky classification and operative procedure on acetabulum revisions

Paprosky’s class	IIa	IIb	IIc	IIIa	IIIb	Cases, *n*(%)
Patients, n (%)	18 (31.0%)	13 (22.4%)	7 (12.1%)	9 (15.5%)	11 (19.0%)	58 (100%)
*Acetabular procedure, n*						
3D-printed TT cup + augment	17	12	4	7	8*	48 (82.8%)
Jumbo cup	1		1			2 (3.4%)
Allograft + TM cup		1	2			3 (5.3%)
3D‑printed triflange cup					1*	1 (1.7%)
Double TM cups				1	1*	2 (3.4%)
IBG + Cemented cup				1*		1 (1.7%)
Cup-cage construct					1*	1 (1.7%)

*Including one case with pelvic discontinuity

Demographic data for the 43 patients are described in Table 2. The indications for revision were aseptic loosening in 31 hips, progressive osteolysis in 5 hips, severe acetabular erosion after hemiarthroplasty in 3 hips, and 4 were the second stage of a two-stage revision for infection. All patients with aseptic failure reported symptoms of hip pain. The mean time elapsed from primary surgery to this revision was 9.4 (range 2.3–17.1) years.

**Table 2:** Characteristics and demographics of the 43 patients who underwent standardized 3D‑printed TT cups and augments

*Gender, n (%)*	Value
Male	16 (37.2%)
Female	27 (62.8%)
*Average age at revision surgery, years (range)*	62.6 (44–77)
*BMI, kg/m*^*2*^* (range)*	24.7 (18.1–37.6)
*Primary diagnosis, n (%)*	
Avascular necrosis of femoral head	16 (37.2%)
Femoral neck fracture	11 (25.5%)
Osteoarthritis	9 (20.9%)
Developmental dysplasia of the hip	3 (7.0%)
Rheumatoid arthritis	2 (4.7%)
Acetabular fracture	2 (4.7%)
*Previous revision, n (%)*	
None	28 (65.1%)
1	11 (25.6%)
2	3 (7.0%)
3	1 (2.3%)
*Reason for revision, n (%)*	
Aseptic loosening	31 (72.1%)
Osteolysis	5 (11.6%)
Acetabular erosion	3 (7.0%)
Revision for infection	4 (9.3%)

### Preoperative preparation

The standardized 3D‑printed TT cups and augments (AK Medical, China) were manufactured by electron beam melting (EBM) technology and were composed of titanium particles melted layer by layer with a three-dimensional mesh structure. The pore size (600–800 μm) and porosity (80%) were determined with computer-aided design.

Computed tomography (CT) was performed to provide 3D structural information on acetabular bone deficiencies, and a 3D-printed pelvis model was established based on the CT data. Then, a simulated surgery was performed on the 3D model, and the preliminary decision of prosthesis selection, prosthesis sizing and the position of augmentation were made.

### Surgical procedure

During the operation, the degree of the acetabular bone defect was confirmed and reassessed by comparison with the 3D-printed hip model. All 43 patients were operated on using a standard posterolateral approach. Extended trochanteric osteotomy (ETO) was performed in seven cases to remove the well-fixed femoral component because of malalignment of the stem (2 cases), leg length discrepancy (2 cases), impingement (1 case), and unacceptable abrasion or corrosion at the head-neck taper of the stem (2 cases). The other four patients underwent ETO for hip stiffness to assist exposure. After removal of the previous acetabular components, the lead surgeon debrided the remaining acetabulum. Synovial fluid and homogenate of the deep tissue specimens were obtained for all patients and were sent to the institutional laboratory for culture. For all patients with a positive culture, intravenous antimicrobial therapy was initiated to cover pathogens in accordance with the culture. The acetabulum defect was carefully and gently reamed from a smaller size to a diameter that was one millimeter or two millimeters less than the planned size to mesh with the augment tightly. The standardized 3D‑printed TT augment was inserted into the acetabular defect and was additionally fixed to the pelvis with 6.5-mm screws. Whenever possible, these screws were placed in the direction of the transmission of load, directly into the cranial ilium. After implantation and fixation of the augment, the acetabulum was reamed with the planned degrees of inclination and anteversion in successive increments from a smaller size to a diameter that was one millimeter or two millimeters less than the planned size. Post-reaming, the grinding debris was removed and repeatedly flushed. Morsellized allogeneic bone graft was used to fill the remaining defect. After that, a trial cup was tested and evaluated by intraoperative fluoroscopy. If the cup position, stability and contact area with the host bone were thought to be acceptable, the augment–cup interface was coated with polymethylmethacrylate cement. Then, a cementless standardized 3D‑printed TT cup was secured and impacted with an adequate press-fit and screws. Finally, the stability of the cup was evaluated again. If the femoral stem was loosening, the taper was significantly worn or osteolysis was present in the proximal femur, it was simultaneously revised. For the one case with pelvic discontinuity, a buttress was also used to construct the posterosuperior acetabular wall.

### Postoperative rehabilitation and follow-up

In general, patients were encouraged to walk with crutches, and partial weight-bearing was allowed on the first day after revision surgery. A full weight-bearing gait was permitted after clinical and radiological review at 6 weeks postoperatively. The patients were followed at regular intervals (6, 12, 24 weeks and annually thereafter). Standard anteroposterior (AP) and lateral radiographs were taken at three postoperative days and routinely at every visit.

### Radiological assessment

Overall survival was defined as remaining free of any further revision of the acetabular component. The inclination and anteversion of the acetabular cup were measured on the AP radiograph based on the method of Bachhal et al. [14]. The Lewinnek safe zone [15] was used to assess the cup position. The criteria for osseointegration of uncemented acetabular components described by Moore et al. [16] were used to assess ingrowth. Loosening of the acetabular component was recognized by a change in its position. More than 3 mm migration of either horizontal or vertical orientation, radiolucent lines of 2 mm or more in all DeLee and Charnley zones [17], fracture of screws or variation in cup angle greater than 5° indicated radiographic failure. Radiographs were screened for progressive radiolucent lines (RLLs). The COR was measured on the preoperative and postoperative AP radiographs based on the modified method of Ranawat et al. [18]. The leg length discrepancy (LLD) was described as the discrepant distance from the base of the teardrop to the corresponding tip of the lesser trochanter.

### Clinical assessment

Clinical evaluation was performed using the Harris hip score (HHS) and Oxford hip score (OHS). Health-related quality of life was assessed using the EuroQol five-dimensional questionnaire (EQ-5D).

### Statistical analysis

All radiographs were reviewed by two of the authors (LJH and PFW) and were completed twice by each of the reviewers at least 3 weeks apart. Inter- and intra-observer reliability was assessed by intraclass correlation coefficients (ICCs) for the measurements. For all radiographic parameters, the mean correlation coefficient of the interobserver reliability was 0.88 (range, 0.83–0.94), and the mean correlation coefficient of the intraobserver reliability was 0.90 (range, 0.77–0.97).

Statistical analysis was performed using SPSS software (version 24, IBM Inc., Armonk, New York). Demographic data were described in means (with ranges) and standard deviation (SD). Categorical variables were described with percentages. Statistical tests performed included Wilcoxon signed-rank test and Mantel–Cox log-rank test. Statistical significance was defined as *p* < 0.05. The cumulative probability of remaining free of revision surgery was assessed with a Kaplan–Meier analysis, and Mantel–Cox log-rank test was used to compare the survival rate.

## Results

### Revision with 3D‑printed TT cups and augments

**Fig. 2:**
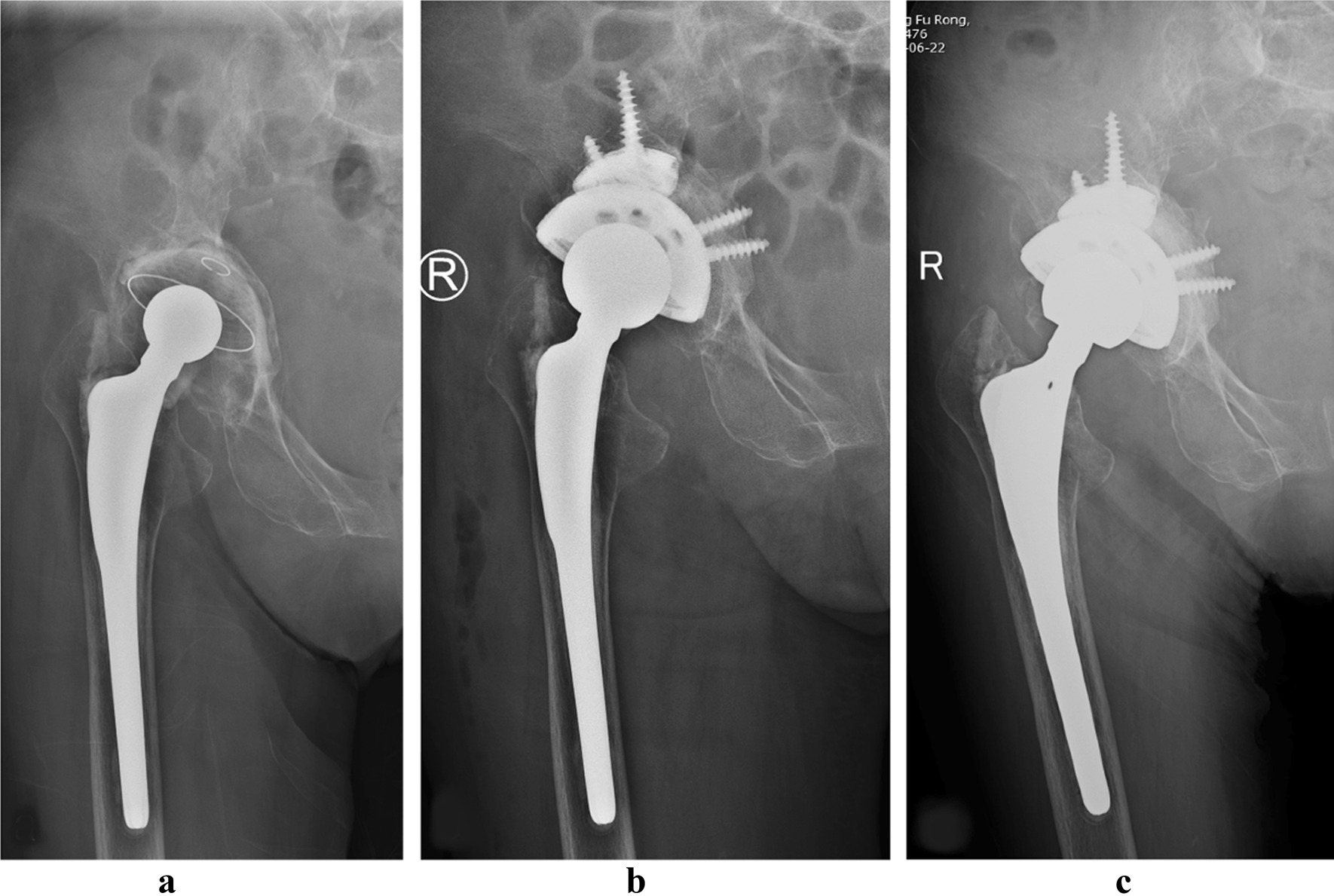
AP radiograph of an 82-year-old female patient with a type IIIB bone defect revised for aseptic loosening of the acetabular cup. **a** before surgery; **b** three days after revision surgery; **c** 72 months after revision surgery

The mean follow-up was 64.5 (range 49–84) months, with a minimum follow-up of 4 years. Acetabular revision was performed as a separate procedure in 13 (30.2%) cases (Fig. 2).

#### Radiological assessments

**Fig. 3:**
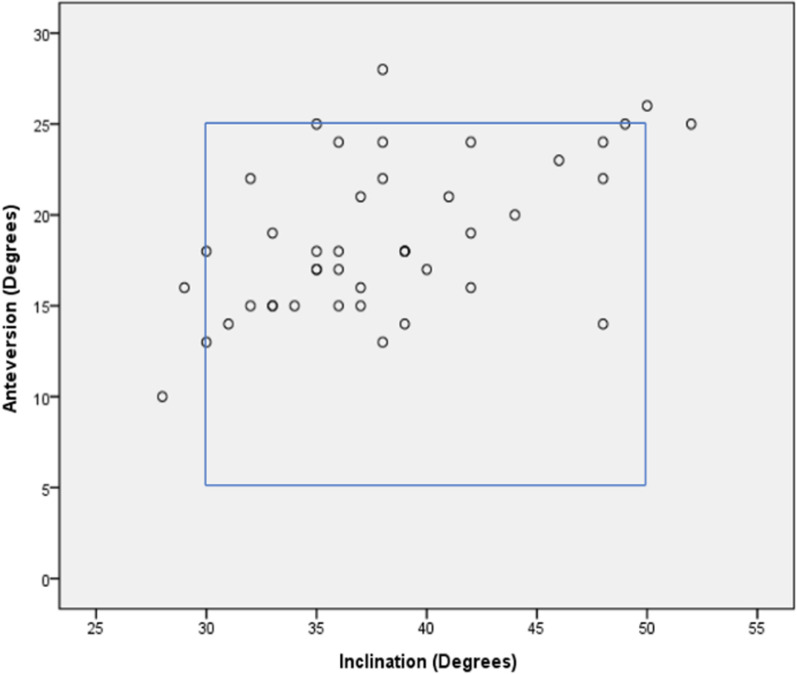
Diagram depicting the cup inclination and anteversion and the number of patients who were within the Lewinnek safe zones

The mean cup inclination was 38.1° ± 6.0° (range 28°–52°), and anteversion was 18.7° ± 4.2° (range 10°–28°). Thirty-eight (88.4%) patients were positioned within the safe zone (Fig. 3).

For the vertical center of rotation (VCOR), the mean value of the surgical side was significantly improved from 42.4 ± 9.1 (range 29–76) mm preoperatively to 22.8 ± 3.4 (range 17–31) mm postoperatively (*p* < 0.001). Although the position of VCOR was obviously corrected, there was still a deviation in the VCOR between the contralateral side (18.9 ± 2.3 mm; range 15–25) and surgical side postoperatively (*p* < 0.001). For the horizontal center of rotation (HCOR), the mean value of the surgical side was 31.2 ± 6.3 (range 10–44) mm preoperatively and 31.5 ± 4.1 (range 22–40) mm postoperatively, with no significant difference (*p* = 0.622). The mean value of the contralateral side was 33.0 ± 3.8 (range 24–39) mm. There was no significant difference between the surgical side and contralateral side preoperatively (*p* = 0.080) and postoperatively (*p* = 0.075).

The mean value of LLD was significantly improved from 38.4 ± 10.7 (range 11–56) mm preoperatively to 4.1 ± 3.0 (range 0–16) mm postoperatively (*p* < 0.001). But in one case, the LLD exceeded 10 mm.

Non-progressive RLLs were observed in three (7.5%) acetabular components with no indications for revision. Of these, one patient showed a radiolucent line in zone I, and two had a radiolucent line in zone III. Radiographic evidence of osseointegration (minimum of three radiological signs) was demonstrated in 36 acetabular components.

#### Clinical assessments

At the latest follow-up, the mean HHS, OHS and EQ-5D scores were significantly improved from 33.0 ± 10.7 (range 11–52), 11.4 ± 3.4 (range 0–21) and 0.29 ± 0.09 (range 0.13–0.41) preoperatively to 80.3 ± 8.8 (range 46–92), 35.8 ± 2.4 (range 26–48) and 0.71 ± 0.10 (range 0.52–0.91) postoperatively (*p* < 0.001). Patient satisfaction reached 93.0% (40/43).

#### Complications

**Fig. 4:**
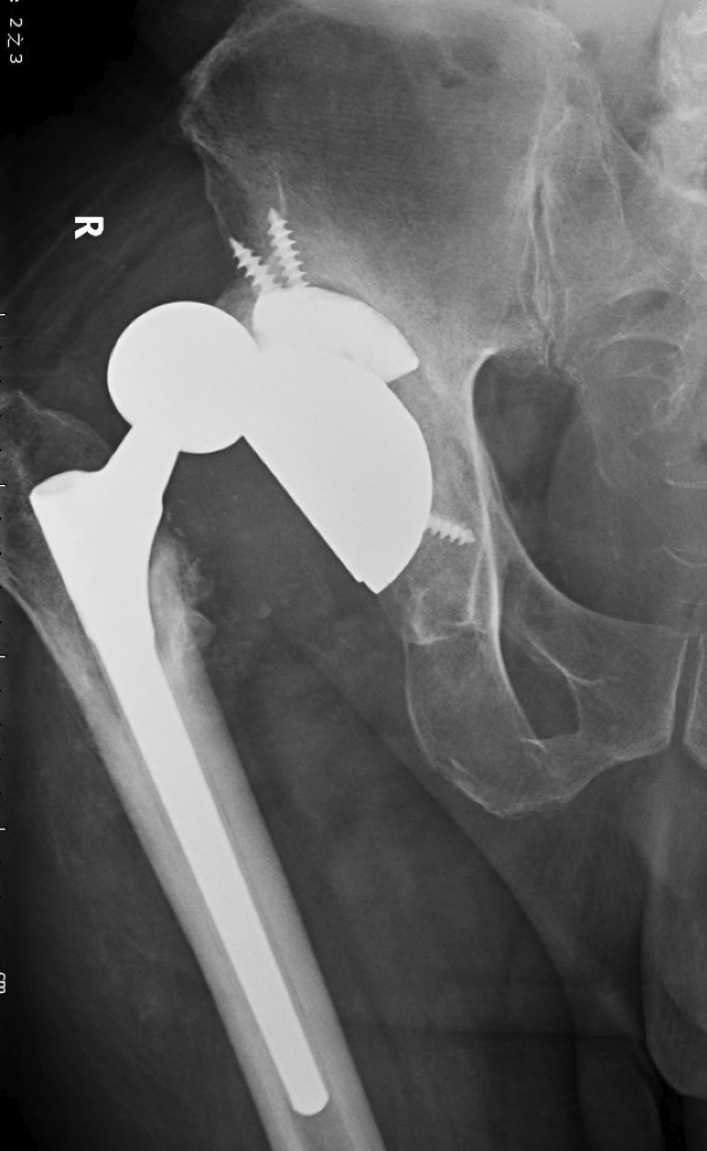
A 66-year-old male patient with dislocation at seven weeks postoperatively

During the surgical procedure, one patient had a fracture of the greater trochanter, and another suffered a split fracture of the femoral shaft around the stem tail. Both patients were treated with osteosynthesis (wire) and achieved bone union. In one case, prolonged wound secretion was noted and healed after debridement. One dislocation (Fig. 4) occurred in a patient after seven weeks that needed closed reduction and external brace fixation, and no recurrent dislocation occurred during the follow-up.

#### Acetabular survivorship

**Fig. 5:**
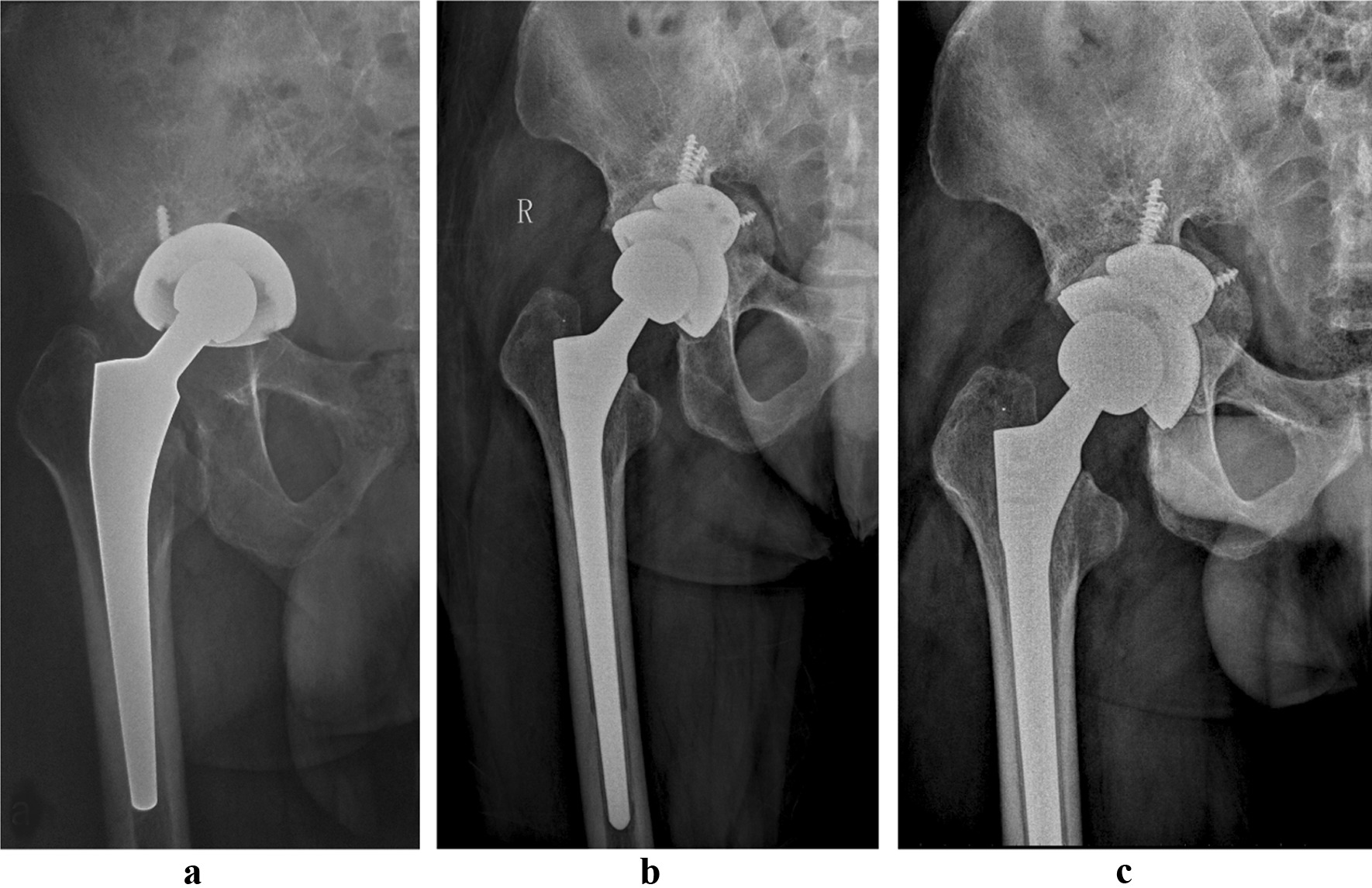
AP radiograph of a 57-year-old male patient with a type IIC bone defect revised for aseptic loosening. **a** before surgery; **b** three days after revision surgery; **c** X-ray with radiolucent lines in the three zones and aseptic loosening of the cup at 36 months postoperatively

There were four revisions (9.3%) in the postoperative period: one for aseptic loosening of the acetabular cup, two for infection and one for periprosthetic femoral fracture. One patient with a type IIC bone defect received further revision at 31 months postoperatively due to aseptic loosening of the acetabular cup (Fig. 5). During revision, the acetabulum was gently reamed upward to increase the contact area between the bone and acetabular components, and a new 3D‑printed TT augment and cup were subsequently inserted. One patient with a type IIIA defect received a two-stage revision due to PJI. One patient with a type IIIB bone defect received a two-stage revision due to recurrence of periprosthetic joint infection (PJI). One patient had further revision of the femoral component due to periprosthetic femoral fracture. However, no acetabular periprosthetic fracture, neurovascular damage or deep venous thrombosis (DVT) was found in our study.

**Fig. 6:**
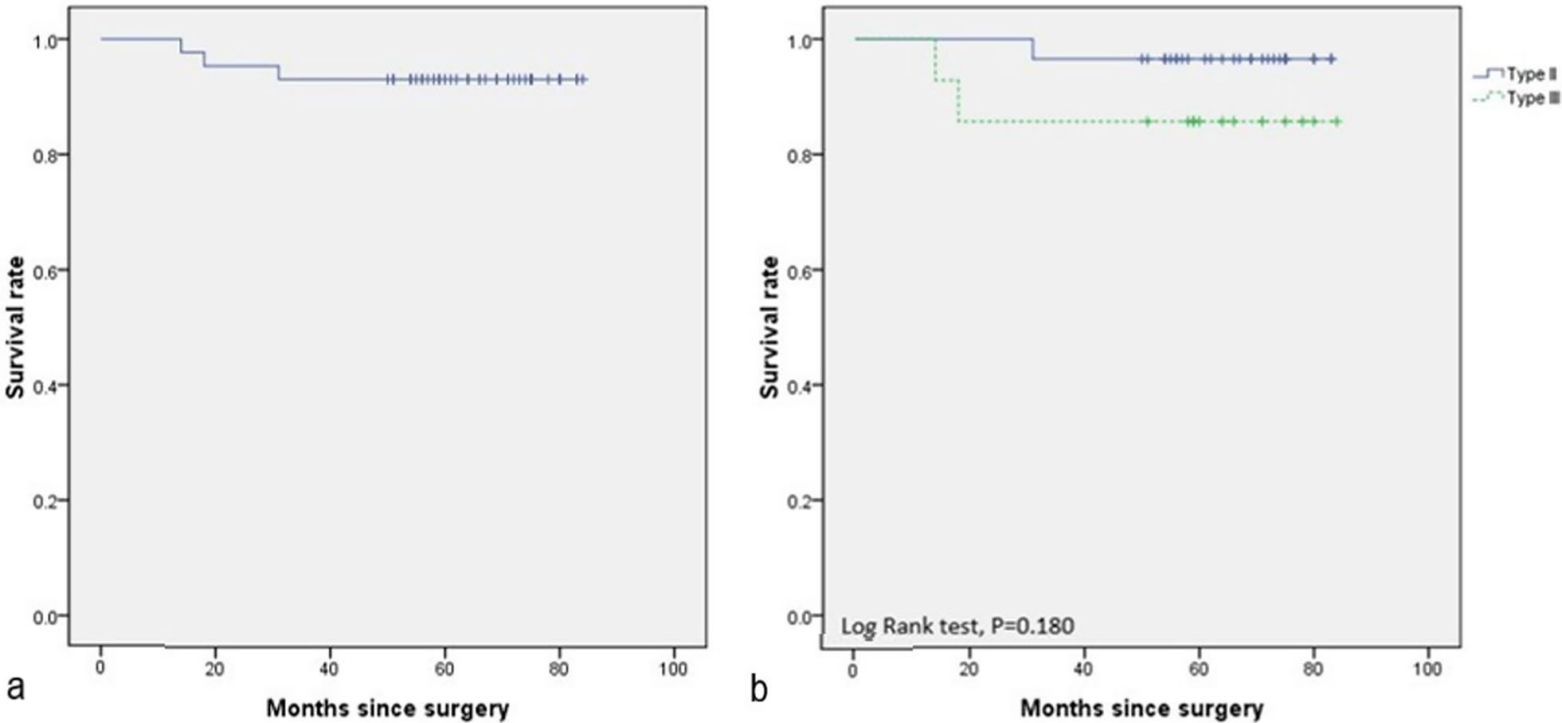
Kaplan–Meier survivorship curve. **a** Kaplan–Meier survivorship with further revision of acetabular component as the endpoint; **b** Kaplan–Meier survivorship according to the Paprosky classification (type II and type III) of preoperative acetabular defect. Log-rank tests were applied to compare survival curves

The revision-free survival rate of the acetabular component at 64.5 months postoperatively was 93.0% (40/43) (Fig. 6). The cumulative probability survival rate between Paprosky type II and type III had no statistically significant difference (*p* = 0.180). With revision for aseptic loosening of the acetabular component as the endpoint, the mean survivorship was 97.7% (42/43). The mean acetabular component survival was 79.6 (95% confidence interval: 74.8–84.4) months, whereas the median survival was unreached in our population.

### Revision with other techniques

Of the 10 patients who underwent revision without 3D-printed cups and augments, one patient who underwent revision using a jumbo cup was lost to follow-up, and another patient who underwent IBG combined with a cemented cup died during the follow-up without undergoing further revision. One patient who was treated using an allograft combined with a TM cup and another patient who was treated using double TM cups failed due to aseptic loosening.

## Discussion

With the development of radiology and 3D printing technology, 3D-printed TT acetabular components have been widely used [8–11]. With a porosity of 80%, a tridimensional trabecular structure, an increased coefficient of friction and excellent biocompatibility, 3D-printed TT augments and cups can possess remarkably fast and complete osseointegration [19]. Moreover, the cup was manufactured using one-piece molding with no risk of coating shedding. The customized 3D-printed TT augment is based on a digital 3D-reconstructed model of the pelvis from computerized tomography (CT) data. However, the slice of the CT scan, the existence of metal artifacts and the capability of engineers to identify effective bone mass will affect the accuracy of the components [20]. Standardized components without a customization cycle are clinically validated, off the shelf and less expensive. According to a systematic review published in 2020 [21], among the six studies that reported revision hip arthroplasty, conventional augments were used in 83.1% (300 out of 361 cases) of their patient cohort. Between 2015 and 2018, more than 80% (48/58) of the revision hips with Paprosky type II and type III acetabular bone defects were performed using standardized 3D-printed TT augments and cups in our institution, and the frequently used augments are concentrated in a few specifications. However, augment as well as buttress was used in only one of the five patients with pelvic discontinuity. Therefore, standardized 3D‑printed TT augments and cups could be used to reconstruct the majority of Paprosky type II and III acetabular defects in revision hip arthroplasty. However, in the presence of pelvic discontinuity, other metallic reconstruction methods are usually required.

To our knowledge, there are few medium-term follow-up studies on the use of 3D-printed TT augments to restore acetabular bone defects. Fang et al. [9] reported the use of patient-specific 3D-printed titanium augments and shells for 24 Paprosky type III acetabular bone defects and customized 3D-printed flanged components for 11 type IIIB patients in revision hip arthroplasty. The survival rate was 100%, with a mean follow-up of 41.5 months. However, a 2-mm cranial migration of the acetabular component was observed on the radiograph at the one-month follow-up. Kong et al. [10] reported that a customized 3D-printed titanium augment with a tantalum trabecular cup was used in the treatment of 23 revision hips with Paprosky type III acetabular bone defects. No re-revision was performed during the mean follow-up of 56 months. In this study, we reported an acetabular component survival rate at 64.5 months of 93.0% in 43 revisions (29 Paprosky type II and 14 Paprosky type III), with a rate of revision for aseptic loosening of 2.3% (1/43). In addition, according to the Paprosky classification of preoperative acetabular defects, the survival rate between Paprosky type II and type III bone defect revisions was not significantly different. Although we had two cases (4.7%) with PJI, it had to be emphasized that we included patients undergoing two-stage revision for septic loosening. Furthermore, we performed ETO in 11 cases (25.6%), which reflects the degree of complexity of these procedures and is a possible risk factor for infection that should be considered. Regardless of infection, the survival rate of the two above studies is still slightly higher than ours. However, with the extension of the follow-up period, the two above studies may be associated with higher failure rates. Moreover, these results are better than those reported for structural bone grafting [7] and reconstruction cages and rings [5, 6].

For a study using the Delta TT system (TT cup with the addition of a hemispherical module) in revision surgery, two-level IV studies reported 88.9% 4-year survival [22] and 98.8% 4-year survival (but with 3/81 migrations, without revision) [23]. Steno et al. [24] even reported that a fatigue fracture of the hemispherical module occurred in the revised case. Our result for aseptic loosening rate was similar or superior to these results. For the tantalum acetabular components, recent studies have shown encouraging medium- to long-term results, with survivorship between 91 and 97% [25–27]. According to Ayers et al. [28], there was no significant difference in proximal migration between tantalum and titanium acetabular cups over a 5-year follow-up period in their series. Meanwhile, the registry results also showed no advantage of tantalum cups compared to titanium [29]. In this study, the reconstruction survival rate (93.0% at 64.5 months) was also comparable to that with tantalum implants [24, 25]. Nonprogressive RLLs were observed in three (7.5%) acetabular components with no migration or revision. Thirty-six of 40 (90.0%) nonrevised acetabular cups were well osseointegrated with 3 or more signs of osseointegration according to Moore's criteria [16]. These findings confirm the results of previous studies [30, 31].

Clinically, we noted a significant and enduring increase in HHS and OHS. The HHS and OHS increased from mean 33.0 and 11.4 preoperatively to mean 80.8 and 36.8 postoperatively, respectively. The health-related quality of life also significantly improved, as shown by the EQ-5D score (mean 0.29 vs mean 0.71). Meanwhile, the patients’ satisfaction reached 93.0% at the end of the follow-up. Zhang et al. [11] and Fang et al. [9] reported average HHSs of 69.2 and 86.1, respectively, after reconstruction with a customized 3D‑printed titanium augment and trabecular cup. Cassar et al. [32] reported a mean OHS of 35 after reconstruction of patients with superolateral acetabular defects using flying buttress porous tantalum augment. Schreurs et al. [33] reported an average HHS of 79 after reconstruction with IBG. Abolghasemian et al. [25] reported a mean OHS of 38 after reconstruction of patients with large acetabular bone defects using a trabecular metal augment and trabecular metal shell. These results were similar to those of the present study.

The inward and upward displacement of the COR weakens the strength of the gluteus medius and affects the abducens function of the surgical hip. In addition, it was reported that positioning a component with a high hip center (upward > 10 mm) has been associated with higher revision rates [34]. However, the tension of the gluteus medius was significantly increased with perihip pain when the COR excessively shifted downward and outward. It may also lead to distressing neurologic symptoms caused by sciatic nerve traction. Therefore, the best scenario is that the COR can be physiologically restored. Some studies [9, 35] claimed that customized 3D-printed acetabular components could more precisely restore the hip rotation center. Fang et al. [9] reported that the postoperative VCOR and HCOR of the operated side were 20.8 (SD 2.0) mm and 30.2 (SD 1.6) mm, respectively. Both the postoperative VCOR (19.5 mm, SD 1.2) and HCOR (31.2 mm, SD 1.7) of the contralateral side were significantly different from those of the operated side. Kong et al. [10] reported that the VCOR was displaced 3.7 mm upward postoperatively and the HCOR was displaced 2.7 mm outward postoperatively compared with the contralateral side. In this study, a relatively normal hip rotation center was restored in almost all patients after revision surgery. No significant difference in HCOR between the contralateral side (33.0 mm, SD 3.8) and surgical side (31.5 mm, SD 4.1) postoperatively was found. Although there was still a deviation in the VCOR between the contralateral side (18.9 mm, SD 2.3) and the surgical side (22.8 mm, SD 3.4) postoperatively, the position of VCOR had been obviously corrected. Compared with those studies using customized 3D-printed augments with TM cups [9–11], our results showed a satisfactory restoration of the hip rotation center.

With the successful restoration of COR, the mean LLD was significantly improved from 38.4 (SD 10.7) mm preoperatively to 4.1 (SD 3.0) mm postoperatively. Only one case had an LLD of more than 10 mm after revision, and the patient was suggested to improve LLD-induced lameness with an elevated insole. The length of the surgical lower limb was extended by nearly 40 mm after surgery compared with an LLD of 56 mm before revision hip arthroplasty. However, the tension of the sciatic nerve significantly increased during the operation, with the risk of neurovascular damage. Furthermore, it was reported that the risk of nerve injury was apparently increased if the surgical leg was extended greater than 4 cm [36].

The implant position, especially cup inclination and anteversion, plays an essential role in instability after hip arthroplasty [11]. In 1978, Lewinnek et al. [15] proposed a ‘safe zone’ of cup inclination of 40° ± 10° and anteversion of 15° ± 10° for the acetabular cup to avoid dislocation. In this study, the mean cup inclination was 38.1° (SD 6.0), and anteversion was 18.7° (SD 4.2). Only five patients were not positioned within the safe zone. However, one case within these target values dislocated at 7 weeks postoperatively. Abdel et al. [37] found that 58% (120 of 206) of their cups were within both safe zones for those who dislocated. Esposito et al. [38] noted no distinct safe zone. Therefore, the Lewinnek safe zone for cup inclination and anteversion may be useful but should not be considered an absolutely safe zone. The target zone of cup position may be individual, taking the bony and muscular anatomy, soft tissue balance and tensioning, spinopelvic motion and surgical approach into consideration.

The stability of the acetabular component/augment interface affects the stability of the entire construct [39]. One patient with a type IIIA defect in this study received further revision at 18 months postoperatively due to aseptic loosening of the acetabular cup. The stability of the augment was confirmed at the time of further revision. Wear particles were noticed from the augment–cup interface, and this was considered to be generated by protracted friction between the cup and the augment. The micromotion of the cup was considered due to thinner cement and incomplete daubing. Then, fracture of the cement was prone to occur with these circumstances [40]. The method of fixation between the acetabular component and augment varies in three ways: screw fixation only, cement fixation only and screw plus cement fixation. With screw fixation only, the friction between the acetabular cup and augment would cause ionization reactions and produce metal ions as long as two different materials were used. Beckmann et al. [39] reported that screw fixation is less stable than cement or screw plus cement fixation. In addition, with screw fixation, the friction between the acetabular component and augment produces metal ions as long as two different materials are used. Cement fixation was the most commonly used. However, fatigue fracture easily occurred when the cement was thin or incompletely daubed or contained bubbles and pores. Although no significant difference was noted when a screw was added to the cement fixation [39], the hybrid fixation should be the most reliable. However, simultaneous screw placement through the acetabular component and augment was difficult due to the shelter of the cement and the shorter time window to handle. In addition, the process of drilling through the acetabular component and augmentation for screw placement created a considerable amount of metal particles, which could not be recovered despite intensive flushing. This may have an adverse influence on the biofunctionality (survival) of the endoprosthesis and present deleterious systemic consequences [41]. For now, this has remained controversial. In our opinion, cement fixation is preferred, but hybrid fixation is still an alternate choice if it is possible to simultaneously place the screws through the acetabular component and augment without drilling.

In conclusion, favorable radiologic results and clinical outcomes were obtained with satisfactory survivorship. Our results clearly supported the continued use of standardized 3D-printed TT augments and cups in revision hip arthroplasty. However, there are still some limitations of this study. First, this was a retrospective single-center study without a control group. In our institution, only 10 revision hips with Paprosky type II and type III acetabular bone defects were treated with reconstruction techniques other than augmentation. Among the 10 patients, six different reconstruction techniques were used. Therefore, the patients using other reconstruction techniques are too few and scattered to become the control groups. Second, two patients were lost to follow-up, and three died during the follow-up, preventing us from providing complete radiological and clinical data. Third, due to the limited number of Paprosky type II and type III acetabular defect cases treated in our institution, the number of cases included in this study was not too large. Therefore, further multicenter controlled studies with larger cohorts and longer follow-up periods are definitely required in the future. Finally, for the patient revised for acetabular loosening, the metal ion levels of joint fluid and blood and metal particles were not analyzed in this study, which should be further researched in subsequent studies.

## Data Availability

The data used and analyzed during the current study are available from the corresponding authors on reasonable request.

## Abbreviations

3D: Three-dimensional
TT: Trabecular titanium
VCOR: Vertical center of rotation
HCOR: Horizontal center of rotation
LLD: Leg length discrepancy
RLLs: Radiolucent lines
HHS: Harris hip score
OHS: Oxford hip score
EQ-5D: EuroQol five-dimensional questionnaire
IBG: Impaction bone grafting
TM: Trabecular metal
ETO: Extended trochanteric osteotomy
AP: Anteroposterior
COR: Center of rotation
PJI: Periprosthetic joint infection
DVT: Deep venous thrombosis
CT: Computerized tomography
